# The Facile Synthesis of a Re-Complex Heterogeneous Catalysis System for Enhancing CO_2_ Photoreduction Activity

**DOI:** 10.3390/ijms241311106

**Published:** 2023-07-05

**Authors:** Bo Li, Hang Li, Shiyan Liang, Jiaao Cheng, Xin Zhong, Yifan Chen, Yujie Song

**Affiliations:** Hainan Provincial Key Laboratory of Fine Chemicals, College of Chemical Engineering and Technology, Hainan University, Haikou 570228, China; l1178938050@163.com (B.L.); 20203100907@hainanu.edu.cn (H.L.); 20190411310075@hainanu.edu.cn (S.L.); 20190411310078@hainanu.edu.cn (J.C.); zhongxin20210831@163.com (X.Z.)

**Keywords:** Re complex, homogeneous, fixation, TiO_2_ gel, heterogeneous, positive synergistic effect

## Abstract

*fac*-Re(2,2’-bipyridine)(CO)_3_Cl] (denoted as ReCC) is an efficient molecule-catalyst with high selectivity in the photoreduction of CO_2_ to CO in a homogeneous system. However, the two major drawbacks of Re(I) complexes in the homogeneous system, easy degradation and difficult separation, seriously hinder its development in the field of industrial applications. In this paper, we designed and prepared two different Re-complex fixation systems (denoted as ReCC@TiO_2_-5 wt% and ReCC-TiO_2_-5 wt%) based on TiO_2_ gel via the sensitization method and sol–gel method, respectively. Compared with a pure ReCC complex, both of them exhibited excellent photocatalytic reduction activity. In particular, the sol–gel hybrid system (ReCC-TiO_2_-5 wt%) displayed outstanding positive synergistic effects on the photocatalytic activity and the long durability of the photocatalytic process. A series of characterizations were carried out to explore the probable photocatalytic reduction process mechanism, which provides the theoretical basis and technical support for the Re complex fixation method.

## 1. Introduction

The photoreduction of CO_2_ has huge potential to reduce the global warming problem and provide carbonaceous fuels (CO, methane, methanol, etc.) for meeting energy needs around the world [1,2,3]. Plentiful catalysts can transform CO_2_ into valuable products. However, a series of CO_2_ transform pathways could emerge along with amounts of byproducts during the reaction process, which extremely reduces CO_2_ conversion efficiency. Therefore, the design and preparation of photocatalysts in the CO_2_ high selectivity conversion has become a hot viewpoint in the photocatalytic field [4,5,6].

The rhenium(I) complex is an attractive photocatalyst in homogeneous reaction systems with a high apparent quantum yield and selectivity for the photoreduction of CO_2_ to CO under visible-light irradiation. Substantive prominent works on rhenium(I) complex derivatives by molecular design strategy have been developed in the homogeneous photocatalytic reaction system. In particular, the rhenium(I) bipyridine (bpy) complexes *fac*-[Re(bpy)(CO)_3_(L)] (L = NCS^−^ or Cl^−^), *fac*-[Re^I^(bpy)(CO)_3_(L)]^n+^ (L = Cl (*n* = 0); L = PR_3_ (*n* = 1)), and *fac*-[Re(bpy)(CO)_3_{P(OEt)_3_]^+^ could act not only as a photocatalyst but also as a photosensitizer, and they show highly efficient photocatalytic conversion and selectivity for CO_2_ to CO in a homogeneous system by one-electron reduced (OER) rhenium complexes species based on the mechanism of the triplet metal-to-ligand charge transfer (^3^MLCT) excited state of rhenium complexes [7,8,9,10,11,12]. However, some drawbacks of rhenium complexes, such as difficulty in recovery from the homogeneous system, the weakness of light response ability, and the complicated modification process, have limited their further development [13]. Therefore, it is necessary to adopt reasonable strategies to improve its recycling capacity and enhance its photoreduction performance.

Rhenium complexes could be combined with photoelectric substrate materials to build a heterogeneous photocatalytic system, which is an effective avenue to solve the recycling issue and endow the Re-based composites with a synergistic effect during the photoreduction process. Numerous solid substrate materials could be utilized to immobilize rhenium complexes, such as TiO_2_ [8,13], SiO_2_ [14], CuInS_2_ Quantum dots (QD) [15], K_x_H_(4−x)_Nb_6_O_17_ [16], MOP [17], COF [18,19], CN [20], to form hybrid materials for the photoreduction of CO_2_ to CO in the heterogeneous system. Among them, TiO_2_ semiconductor works as advantageous substrate material and is used widely in the photocatalyst field because of its stable chemical properties, low cost, non-toxicity, and excellent photoelectric physical chemistry performance. Correspondingly, various preparation methods or strategies are accepted to construct TiO_2_-based composites, for example, sensitization, covalent modification, the hydrothermal method, and deposition method. Via facile sensitization, organic–inorganic hybrid materials based on rhenium complexes and TiO_2_ are obtained and exhibit high activity and selectivity during the photocatalytic reduction CO_2_ to CO process with long durability in the heterogeneous system. Anna Reynal et al. reported a heterogeneous catalyst ReP-TiO_2_ hybrid based on a phosphonate Rhenium bipyridine complex (ReP) and TiO_2_ by the sensitization method, displaying a good turnover number (TON) of 48 mol_CO_ mol_Re_^−1^ for CO_2_-reducing in DMF with triethanolamine as the electron sacrificial agent under visible-light irradiation, compared to the homogeneous systems previously reported [13,21,22]. However, the rhenium complex immobilization on the surface of TiO_2_ not only requires high dispersibility of nanomaterials, but also does not easily come off from the surface of TiO_2_ in the sensitization system during the photocatalytic reaction process. In addition, the utilization efficiency of TiO_2_ blocks is low because of the aggregation of TiO_2_ nanomaterials for the sensitization system, which could easily cause the photogenerated charge recombination to reduce the photocatalytic activity. Thus, to promote the adsorption stability of rhenium complexes on TiO_2_ and advance the photogenerated charge separation and transfer between the rhenium complexes and the surface interfacial of TiO_2_ simultaneously, an effective combination strategy should be proposed to construct the uniform and robust organic–inorganic hybrid materials.

The sol–gel method is a facile and effective strategy for acquiring TiO_2_-based composites doped with a series of functional elements or materials (Ag [23], Cu [24,25], Mn [24,26], graphitic carbon [27], N [25], etc.) in the field of CO_2_ photoreduction. The TiO_2_-based composite materials above exhibit enhanced photocatalytic activity and long durability in reducing CO_2_ to fuel because of the positive correlation synergistic effect between the components in the uniform photocatalytic architecture. The introduction of functional elements or materials in TiO_2_-based composites could effectively regulate the energy potential level and position of the semiconductor [28,29,30,31,32]. However, the dopants above have a limited influence on the light response ability and the separation of photogenerated charge, mainly because of the weak light response ability of the dopants themselves and the serious photogenerated charge recombination caused by the introduction of excess defect sites. Thus, the selection and regulation of the structure and composition of dopants are vitally important in the photocatalytic field. Functional materials with excellent optical physical chemistry performance can be obtained via an in situ sol–gel method containing hydrolysis and condensation reactions with the precursor of TiO_2_ [33,34,35,36,37]. According to the previous achievements of our research group in the construction of functional composites [38,39,40,41], the hybrid nanomaterials displayed excellent light response ability and efficient photogenerated charge separation and transfer ability owing to the following advantages. First, the cross-linking of the photosensitizer and TiO_2_ components in the hybrid materials at molecule level resulted in a uniform microporous/mesoporous structure to protect the photosensitizer molecules from falling off from the surface of TiO_2_. Second, the structure and composition of the photosensitizer can be designed and modified to regulate the photogenerated charge separation and migration between the photosensitizer and TiO_2_ semiconductors in the hybrid. At last, in the hybrid, TiO_2_ can act as electron transfer reservoir and mediator to effectively promote the photogenerated charge separation [1,13]. Therefore, in this paper, we prepared a series of hybrid materials by the in situ sol–gel method as heterogeneous photocatalysts, denoted as ReCC-TiO_2_-X (X = 1 wt%, 3 wt%, 5 wt%, 7 wt%, and 9 wt% representing the mass fraction of ReCC in the hybrid) based on a photosensitizer catalyst, the rhenium(I) bipyridine (bpy) complexes [Re(bpy)(CO)_3_Cl] [7,8] (denoted as ReCC, Scheme S1 and Figure S10) and TiO_2_, for photoreduction of CO_2_. The results indicated that ReCC-TiO_2_-5 wt% could selectively photo-reduce CO_2_ to CO with the highest photoreduction activity of 1.014 mmol g^−1^ h^−1^ with 1,3-dimethyl-2-phenyl-1,3-dihydrobenzimidazole (BIH, Scheme S1 and Figure S10) [42] as the sacrificial agent under visible-light (λ > 420 nm) irradiation, compared to the ReCC photocatalyst, TiO_2_, the sensitization system ReCC@TiO_2_-5 wt%, and other ReCC-TiO_2_-X hybrid materials (X = 1 wt%, 3 wt%, 7 wt%, and 9 wt%). Furthermore, the mechanisms of CO_2_ photoreduction for ReCC-TiO_2_-5 wt% were explored.

## 2. Results and Discussion

### 2.1. Morphological and Structural Characterization

**Figure 1:**
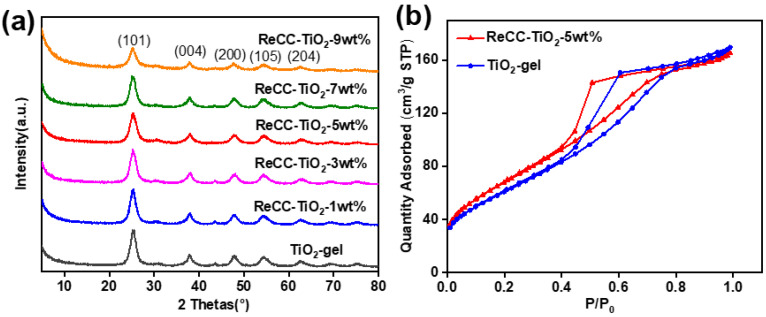
(**a**) The XRD patterns of the prepared photocatalyst samples. (**b**) N_2_ adsorption–desorption isotherms of ReCC-TiO_2_-5 wt% and TiO_2_-gel detected at 77 K.

The X-ray diffraction (XRD) pattern displayed in Figure 1a illustrates the peaks corresponding to the ReCC-TiO_2_ samples with varying ReCC mass contents. The primary characteristic peaks observed were in agreement with the anatase TiO_2_ crystal planes, specifically (101), (103), (200), (105), and (204). These findings indicated the successful formation of anatase TiO_2_ in the hybrid materials, with minimal impact on crystallinity owing to the low doping levels of ReCC. Furthermore, through a comparison between the observed diffraction peaks in our study and the reference peaks listed in the JCPDS (21-1272) standard diffraction cards, clear correlations could be identified between the peaks and different crystal faces. This correspondence further supported the validation of a successful synthesis of composite materials comprising anatase titanium dioxide. In addition, the loading capacity of the Re complex was below the theoretical value, being less than 10%. As a result, the XRD pattern did not exhibit any distinct characteristic peak related to the Re complex, indicating the formation of anatase TiO_2_ in the hybrid materials with little impact on the crystallinity owing to the small doping contents of ReCC.

Moreover, the Brunauer–Emmett–Teller (BET) surface areas and the pore size distributions data for ReCC-TiO_2_-5 wt% and TiO_2_-gel shown in Figure 1b, Figure S1 and Table S1 were measured by N_2_ adsorption–desorption isotherms at 77 K. The adsorption–desorption curve types for ReCC-TiO_2_-5 wt% and TiO_2_-gel are Type IV isotherm lines simulated and calculated by the Barrett–Joyner–Halenda (BJH) method, displaying mesoporous characteristic materials (Figure S1). Compared to the BET values of the TiO_2_-gel (231.3 m^2^ g^−1^) with 4.54 nm mesopores (Figure S1), the calculated BET value of ReCC-TiO_2_-5 wt% with 3.96 nm mesopores (Figure S1) was a slight increase to 258.1 m^2^ g^−1^, which suggested that ReCC and TiO_2_ in the hybrid could be combined by covalent bonds to form multi-porous functional materials through the sol–gel process.

**Figure 2:**
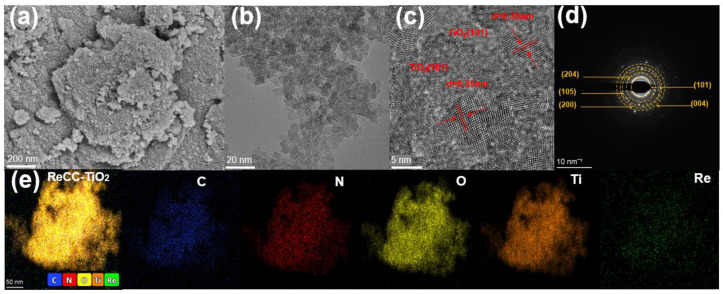
(**a**) SEM, (**b**) TEM, (**c**) HTEM, (**d**) SAED, and (**e**) the elemental mappings image of ReCC-TiO_2_-5 wt%.

The SEM, TEM, SAED, elemental mappings images, and EDX results of the ReCC-TiO_2_-5 wt% photocatalyst are recorded in Figure 2 and Figure S2. In Figure 2a,b, SEM and TEM images investigation provided insights into the fundamental morphology of the material, highlighting the presence of uniformly dispersed ellipsoidal nanoparticles with a size range of 10 to 20 nm. Furthermore, analysis of the HRTEM images for ReCC-TiO_2_-5 wt% indicated that the d-spacing between two adjacent lattice planes was about 0.35 nm (Figure 2c), attributed to the spacing of the TiO_2_(101) plane. The SAED results confirmed the amorphous crystallinity of TiO_2_ for ReCC-TiO_2_-5 wt% (Figure 2d). The EDX results displaying the different contents of C, Ti, O, N, and Re elements are seen in Figure S2, further demonstrating that both the ReCC and TiO_2_ existed in the hybrid. The elemental mapping results showed that C, Ti, O, N, and Re elements were distributed homogenously throughout the whole of the ReCC-TiO_2_-5 wt% materials (Figure 2e), manifesting that the ReCC complex was uniformly combined with TiO_2_ by robust linking bonds in the hybrid. The above morphology analysis indicated the successful synthesis of ReCC-TiO_2_ with a multi-porous structure. Moreover, the elemental compositions and valence states of ReCC-TiO_2_-5 wt% were detected by the XPS test, as shown in Figures S3 and S4. The survey results indicated that C, Ti, O, and Re elements existed in the ReCC-TiO_2_-5 wt% composite. The peaks of 458.8 and 464.5 eV are labeled as the binding energy chemical shift of Ti 2p_3/2_ and Ti 2p_1/2_, respectively, and the peak of 529.9 eV is attributed to the binding energy chemical shift of O 1s, both of which above could be ascribed to the Ti-O bond of TiO_2_ for ReCC-TiO_2_-5 wt%. Moreover, the binding energy at 42.1 and 45.8 eV belonged to Re 4f_5/2_ and Re 4f_7/2_, respectively. The atomic ratio of Re for the ReCC-TiO_2_-5 wt% composite was about 0.08%, suggesting that ReCC complex existed in the hybrid, and that ReCC was successfully doped into TiO_2_ via the sol–gel method.

**Figure 3:**
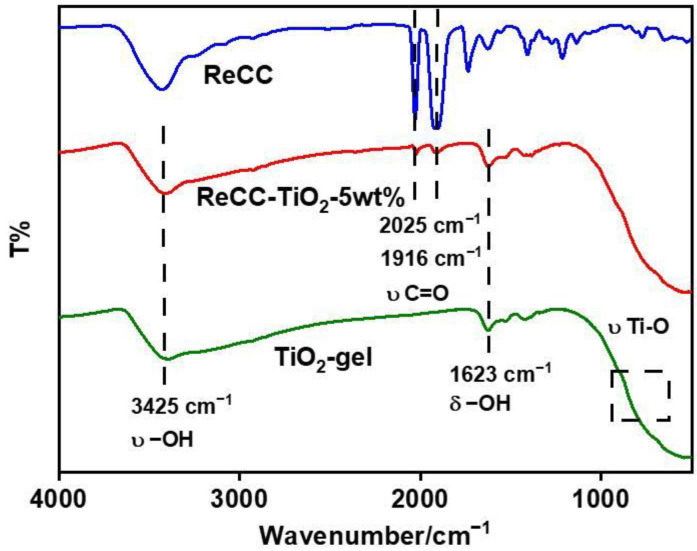
The FT-IR spectra of ReCC, TiO_2_-gel, and ReCC-TiO_2_-5 wt%.

The FT-IR spectra of the ReCC, TiO_2_-gel, and ReCC-TiO_2_-5 wt% samples are shown in Figure 3. The absorption peaks of 2025 and 1916 cm^−1^ for ReCC and ReCC-TiO_2_-5 wt% samples are attributed to the stretching vibrations of C=O and C-O from -COOH group in ReCC, indicating that ReCC was successfully introduced into TiO_2_. The 3425 and 1623 cm^−1^ of absorption peaks belong to the characteristic vibrations of -OH groups from TiO_2_-gel. The existence of the -COOH group in ReCC and the -OH group in TiO_2_-gel could provide an opportunity for the robust combination of ReCC and TiO_2_ at the molecule level in the hybrid. This was verified by the characteristic vibration bands of ReCC-TiO_2_-5 wt%, which appear simultaneously in the structure feature vibration bands above of ReCC and TiO_2_ at 3425, 2025, 1916, and 1623 cm^−1^, indicating the existence of ReCC and TiO_2_ in the ReCC-TiO_2_-5 wt% sample. In addition, for the ReCC-TiO_2_-5 wt% materials, the characteristic vibration peaks of C=C on the benzene ring from ReCC emerge at 1500 cm^−1^ and the bulging characteristic peak of Ti-O from TiO_2_ appears at 500–1000 cm^−1^. Therefore, ReCC could be combined with TiO_2_ through the dehydration condensation of carboxyl from ReCC and hydroxyl groups on the surface of TiO_2_ to facilitate photogenerated charges separation and transfer between ReCC and TiO_2_. Moreover, thermogravimetric analysis (TGA) of ReCC, TiO_2_-gel, and ReCC-TiO_2_-5 wt% was executed in an N_2_ atmosphere with a heating rate of 10 °C/min to acquire the information on their thermostability. The mass loss below 100 °C for ReCC, TiO_2_-gel, and ReCC-TiO_2_-5 wt% was ascribed to the volatilization of H_2_O and solvent. However, a pure ReCC complex could resolve at about 150 °C, suggesting that the thermostability of the Re complex is feeble. This indicated the Re complex could be inactivated easily (Figure S5).

### 2.2. Photochemical Properties and Band Structure

**Figure 4:**
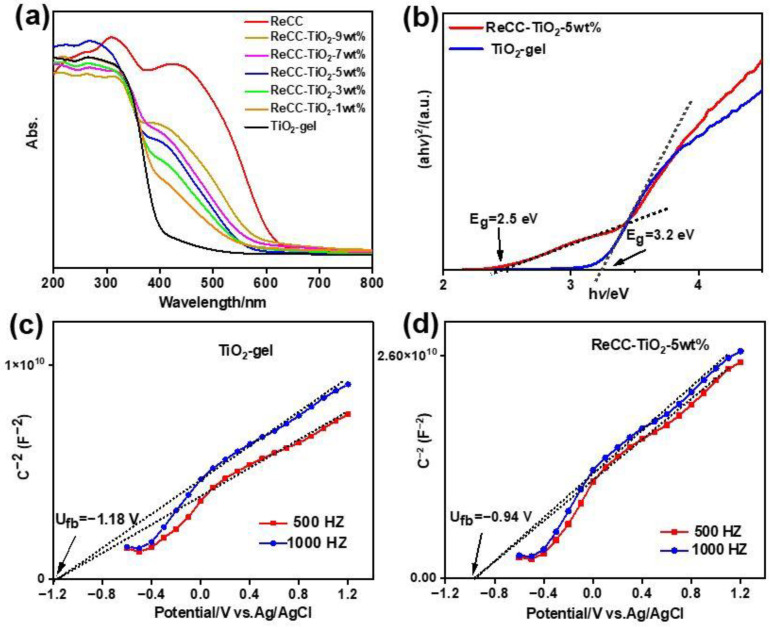
(**a**) UV-vis diffraction spectra of TiO_2_-gel, ReCC, and ReCC-TiO_2_-X (X = 1 wt%, 3 wt%, 5 wt%, 7 wt%, and 9 wt%). (**b**) The Tauc plot of TiO_2_-gel and ReCC-TiO_2_-5 wt%. Mott–Schottky plot of (**c**) TiO_2_-gel and (**d**) ReCC-TiO_2_-5 wt%.

The UV-vis absorption spectra of ReCC, TiO_2_-gel, and ReCC-TiO_2_-X (X = 1 wt%, 3 wt%, 5 wt%, 7 wt%, and 9 wt%) are presented in Figure 4a. Among the investigated materials, the TiO_2_-gel sample exhibits the narrowest light absorption range. However, as the doping amount of the Re complex increased, the absorption range of the composite gradually expanded. This observation confirmed the effective enhancement of the light absorption ability of the composite as the incorporation of the Re complex. The main response region of TiO_2_-gel is at the ultraviolet part (λ < 400 nm) with the maximum adsorption peak at about 320 nm, accordant with the 3.2 eV of band gap (Figure 4b). Compared to TiO_2_-gel, ReCC has a relatively wide absorption range in the region of 200–600 nm with a maximum adsorption peak of 450 nm (Figure 4a), which could be ascribed to a triplet metal-to-ligand charge transfer (^3^MLCT) excited state of ReCC, containing a conjugated π-π* and p-π* structure. For the hybrid ReCC-TiO_2_, the absorption band region is from 200 to 550 nm with a shoulder band at about 400 nm (Figure 4a). With the increase in ReCC, the light absorption intensity of ReCC-TiO_2_ also increased, while a little blueshift is observed for the hybrid, compared to complex ReCC. The possible reason is that carboxylic group of ReCC could react with -OH on the surface of TiO_2_ at the molecular level to form ester groups, which could reduce the push–pull effect on the electronic structure of ReCC itself. In Figure 4b, ReCC-TiO_2_-5 wt% exhibits two band gaps: a wide band gap of 3.2 eV and a narrow band gap of 2.5 eV. The former could be attributed to the band gap of TiO_2_ and the latter is ascribed to the introduction of ReCC in the hybrid. However, the E_0-0_ value of pure ReCC is about 2.2 eV in Figure S6, which is narrower than the band gap of ReCC in the hybrid. This indicated that it was not a simple physical mixture between ReCC and TiO_2_, while the robust linkage formed on the atomic level between ReCC and TiO_2_ in the hybrid. In addition, the smaller band gap not only indicates the wider light spectral response range, but also displays the lower VB position for the composite, while the VB potential for the composite was still higher than the CB edge of TiO_2_-gel and the photoreduction potential of CO_2_ to CO. Therefore, it was beneficial for photocatalytic conversion owing to the electron fast transfer on the gas–solid reaction interface. In the Mott–Schottky plots of TiO_2_-gel and ReCC-TiO_2_ (Figure 4c,d), the positive slopes indicate that all samples are n-type semiconductors. The flat band potentials of TiO_2_-gel and ReCC-TiO_2_ were −1.18 and −0.94 V (vs. Ag/AgCl), respectively. The conduction band (CB) potential of n-type semiconductors is generally nearly equal to the value of flat band potentials (U_fb_). The CB potentials of TiO_2_-gel and ReCC-TiO_2_ were evaluated to be −0.98 and −0.74 V vs. NHE, which demonstrated that the introduction of ReCC could effectively adjust the energy level position and the band gap width of the hybrid. Moreover, the reduction potential of ReCC was about −0.58 V vs. NHE, and the reduction potential of the ferrocene was as a reference, as shown in Figure S7, which was more negative than the redox potential of CO_2_/CO. In addition, ReCC itself could absorb visible light and easily be excited, enhancing photogenerated electrons jumping to the reduction potential of ReCC to realize the photoreduction of CO_2_ to CO. Therefore, in the hybrid, TiO_2_ not only worked as a scaffold or protectant to disperse and immobilize ReCC, but also acted as an electron bank to accept, save, and release electrons to facilitate the photoreduction process of CO_2_ to CO.

### 2.3. Photocatalytic CO_2_ Reduction Activities

The photocatalytic reduction activities of ReCC-TiO_2_-X (X = 1 wt%, 3 wt%, 5 wt%, 7 wt%, and 9 wt%) on the CO generated were estimated by gas chromatography under the visible-light irradiation along with the liquid phase being analyzed by HPLC to confirm the existence of other exclusive products, such as H_2_ or formic acid. Neither of them were detected.

**Figure 5:**
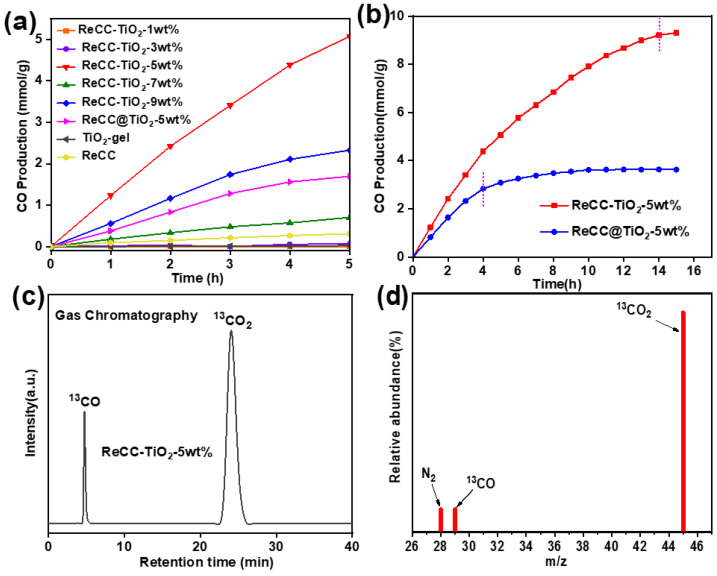
(**a**) The samples of the photoreduction activity for CO production in 5 mL DMF with 134 mg BIH as the electron donor under visible-light irradiation (λ > 420 nm). (**b**) The photoreduction stability tests for ReCC-TiO_2_-5 wt%. (**c**,**d**) GC-MS spectra of ReCC-TiO_2_-5 wt% dispersion in ^13^CO_2_-saturated DMF with BIH as the electron donor under visible-light irradiation (λ > 420 nm) for 5 h.

**Figure 6:**
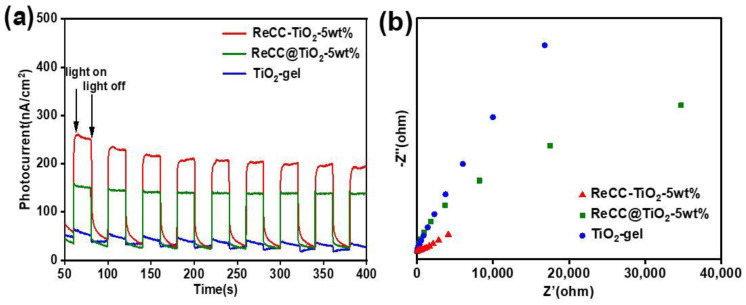
(**a**) Transient photocurrents and (**b**) EIS Nyquist plots of TiO_2_-gel, ReCC-TiO_2_-5 wt%, and ReCC@TiO_2_-5 wt% in Na_2_SO_4_ aqueous solution.

An isotope-labeling test was performed for ReCC-TiO_2_-5 wt% dispersions in a ^13^CO_2_-saturated DMF system with BIH as the sacrificial agent. In Figure 5c,d, the resonance of the ^13^C isotope for ^13^CO was analyzed by GC-MS and revealed at the retention time of 4.9 min with the *m*/*z* peak of 29. Moreover, in Figure 5a, ReCC-TiO_2_-1 wt% displays only a CO production activity of 0.000268 mmol g^−1^ h^−1^. With the mass content ratio increase in ReCC to 5.0 wt%, the best photoreduction CO production rate was about 1.04 mmol g^−1^ h^−1^. Then, the photocatalytic activity of ReCC-TiO_2_ decreased in sequence as the mass percentage of ReCC further increased, indicating that the photoreduction activities of the hybrid were not just a positive correlation with the complex-doped contents. It is manifest that the excessive ReCC complex might impede the separation and transfer of the photogenerated. Besides, the contrast experiment, the CO production activity of ReCC-TiO_2_-5 wt% dispersions in an Ar-saturated DMF system is almost little (Figure S8), further suggesting that the resource of CO is CO_2_. Furthermore, the photoreduction activity of pure ReCC and TiO_2_-gel were only 0.0628 and 0.00495 mmol g^−1^ h^−1^, respectively, as shown in Figure 5a. Compared with the photocatalytic activity of ReCC in a homogeneous reaction system or TiO_2_-gel in a heterogeneous system, the introduction of ReCC into TiO_2_ greatly enhanced the photoreduction activity of CO_2_. To investigate the influence of different preparation methods on the photocatalytic performance in this paper, the CO generation rate of the ReCC@TiO_2_-5 wt% surface sensitization system was evaluated to 0.339 mmol g^−1^ h^−1^, three times lower than the ReCC-TiO_2_-5 wt% catalysts, which is ascribed to not only the increase in Re complex doping contents, but also the robust coordination between ReCC and TiO_2_ in the hybrid. Furthermore, the photo-durability performance of ReCC-TiO_2_-5 wt% and ReCC@TiO_2_-samples under long-time visible-light irradiation was evaluated, as shown in Figure 5b. The photocatalytic CO production rate of ReCC-TiO_2_-5 wt% tended to flatten after 14 h, while ReCC@TiO_2_-5 wt% was deactivated only after 4 h. Combined with the results of ICP-AES, the loading amounts of ReCC in ReCC-TiO_2_-5 wt% were higher than that of ReCC@TiO_2_-5 wt%, indicating that the robust coordination binding bond existed between ReCC and TiO_2_ in the hybrid ReCC-TiO_2_-5 wt%. In the ReCC@TiO_2_-5 wt% system, the ReCC complex easily fell off from the TiO_2_ surface, implying that the weak binding approaches existed between ReCC and TiO_2_. This was further verified by EIS measurements seen in Figure 6b. Compared to the sensitization system, the ReCC-TiO_2_ composite had less charge transfer resistance between the interface of ReCC and TiO_2_. Therefore, the sol–gel method could give full play to the synergistic effect on rhenium complexes and titanium dioxide and effectively enhance the stability of the hybrid material. This showed the result of the TON_CO_ (representing the mole numbers of CO production versus the mole amount of ReCC doped in the hybrid) for the ReCC-TiO_2_-5 wt% sample with a total irradiation time of 14 h, giving a high TON_CO_ of 246.2 with no significant falling-off.

### 2.4. Charge Transfer Process and Photocatalytic Mechanism

Why would the different preparation methods in this work greatly enhance the photocatalytic CO_2_ reduction performances? The photoelectrochemical measurements were executed to manifest the interface charge behaviors (Figure 6). The transient photocurrent plots for TiO_2_-gel, ReCC-TiO_2_-5 wt%, and ReCC@TiO_2_-5 wt% are shown in Figure 6a, and the results demonstrated that ReCC-TiO_2_-5 wt% synthesized by the facile sol–gel method presented a higher photocurrent density compared with the counterpart, ReCC@TiO_2_-5 wt% obtained by the sensitization method. Further compared with the pure TiO_2_-gel sample, ReCC-TiO_2_-5 wt% also exhibited a higher photocurrent response intensity. It demonstrated that the introduction of ReCC into the hybrid materials enhanced the visible-light response ability and facilitated the photogenerated charges separation, transfer, and enrichment to further achieve the CO_2_ photoreduction on the interface between ReCC and TiO_2_. As seen in Figure 6b, the EIS measurements of the samples, TiO_2_-gel, ReCC-TiO_2_-5 wt%, and ReCC@TiO_2_-5 wt%, were carried out with an electrochemical analyzer to evaluate the internal resistance performance during the charge separation and migration process. Notably, in a comparison between the parent TiO_2_-gel and the sensitization sample ReCC@TiO_2_-5 wt%, ReCC-TiO_2_-5 wt% showed the smallest semicircular diameter of the Nyquist plots, indicating the smallest charge-migration resistance and suggesting that the photogenerated electron separation and transfer efficiency with the lowest impedance occurred at the interface between ReCC and TiO_2_ in the ReCC-TiO_2_-5 wt% composite.

The different mechanism pathways in CO_2_ photoreduction by [Re^I^(bpy)(CO)_3_L] derivative catalysts have been proposed and discussed in the aforementioned published work [1,43,44]. A possible route is the charge separation and transfer processes of ReCC-TiO_2_ for the significantly high selectivity on the reduction of CO_2_ to CO (Figure S9). According to the light response capacity and the redox potential values of ReCC and TiO_2_-gel (Figure 4 and Figure S7), we can tell that ReCC works as light absorption units and catalytic centers in the CO_2_ reduction reaction, while TiO_2_ in the hybrid not only acts as a stabilizer for supporting and protecting the Re complex, but also works as an electron reservoir to accept electrons from the excited states of ReCC (ReCC*) and then easily releases electrons to ReCC for photoreduction of CO_2_ to CO in dynamics. Therefore, the possible mechanistic details of CO_2_ reduction in this hybrid are described as below (Figure S9): Under visible-light irradiation, the ReCC complex in the hybrid was excited to become Re^I^CC* along with the transition of electrons from the HOMO orbital to the LUMO orbital. Then the excited electrons on Re^I^CC* transferred to the CB of TiO_2_ in the hybrid to be stored. After the electron transfer, the Re^I^CC* transformed into a Re^II^CC^+^ cation, which easily combined with DMF to form Re^II^CC^+^(DMF) intermediates. The intermediates underwent the reversible exchange adsorption reaction with CO_2_ molecular to become the CO_2_-coordinated complex Re^II^CC^+^(CO_2_). With the polarization of water molecules, the Re^II^CC^+^(COOH) formed by carboxylation was easily inclined to accept electrons from the CB of TiO_2_ to facilitate the reduction of the -COO- and H^+^ groups of Re^II^CC^+^(COOH) in the hybrid ReCC-TiO_2_, which was verified by the formation of the main amounts of CO and trace amounts of H_2_. Since the photoreduction of CO_2_ to CO by ReCC complex is a multi-electron process, it is essential for the enrichment and storage of electrons on the surface of TiO_2_ in the hybrid. However, little H_2_ production was attributed to the reduction in H_2_O adsorbing on the surface of the hybrid materials or existing in the solvent. At last, the Re^II^CC^+^ was reduced to Re^I^CC by the sacrificial agent BIH for the recycle.

## 3. Materials and Methods

### 3.1. Materials

All chemical agents used in this paper were directly utilized without any further treatment. The main chemical raw materials, 2,2′-Bipyridyl-4,4′-dicarboxylic acid (>99%), rhenium pentacarbonyl chloride (>99%), and titanium butoxide (>99%) were supplied from Sinopharm Chemical Reagent Co. Ltd (Shanghai, China). BIH and ReCC were obtained as in previously mentioned literature [36,37]. The synthesis processes are listed in Scheme S1. The main gases ^13^CO_2_ and CO_2_ were provided by Yuejia Gas Co., Ltd. (Guangzhou, China).

### 3.2. Preparation of Photocatalyst

#### 3.2.1. The Preparation of ReCC-TiO_2_ Photocatalyst

The ReCC-TiO_2_ composites were prepared by the sol–gel method. To acquire ReCC-TiO_2_-5 wt% composite, acetic acid (170 μL), distilled water (100 μL), THF (1 mL), and butyl-titanate (1 mL) were subsequently added into a sample bottle, and ReCC (2.5 mg) (Scheme S1) in THF was added into the above bottle and stirred until it became a transparent dark yellow sol solution, then it became dry transparent gel overnight. The dry gel material was washed and hydrolyzed with distilled water at 100 °C for 48 h and then evaporated at 45 °C to dryness to obtain light yellow solid powder. The doping mass ratio of ReCC in the hybrid detected by ICP-AES was 0.70 wt%. Similarly, ReCC-TiO_2_-X (X = 1 wt%, 3 wt%, 7 wt%, and 9 wt%) and TiO_2_-gel without ReCC were acquired by the above process. The doping mass ratios of ReCC in the hybrid ReCC-TiO_2_-X (X= 1 wt%, 3 wt%, 7 wt%, and 9 wt%) were 0.11 wt%, 0.37 wt%, 1.1 wt%, and 1.8 wt% successively.

#### 3.2.2. The Preparation of the ReCC@TiO_2_-5 wt% Photocatalyst

The TiO_2_-gel prepared with the above method was dispersed in a 3.125 mM THF solution of ReCC overnight, and then the residues were centrifuged and washed with THF and water until the eluent observed was no longer colorful. The solid was dried at 45 °C overnight. The loading mass ratio of ReCC in ReCC@TiO_2_-5 wt% detected by ICP-AES was 0.5 wt%.

### 3.3. Characterization

The ^1^H spectra were recorded on a Bruker AVANCE NEO 400. The absorption spectra were observed with a Shimadzu UV-2600 (Shimadzu, Kyoto, Japan) spectrometer and the fluorescence spectra were measured with HORIBA FluoroLog-3 (Jobin Yvon Inc., Newark, NJ, USA). Powder X-ray diffraction (PXRD) was recorded on a Rigaku Smart Lab diffractometer (Rigaku, Beijing, China) (Cu-Ka1 radiation, λ = 1.54056 Å). Transmission electron microscopy (TEM) images, high-resolution transmission electron microscopy (HRTEM) images, high-angle annular dark field scanning transmission electron microscopy (HAADF-STEM) micrographs, selected area electron diffraction (SAED) data, energy dispersive X-ray spectroscopy (EDX) data, and elemental mapping results were obtained using an FEI Talos F200X transmission electron microscope (FEI, Hillsboro, OR, USA) at 200 kV. Scanning electron microscopy (SEM) micrographs were recorded on a Gemini300 microscope (ZEISS, Jena, Germany). Gas adsorption measurements were performed on an ASAP 2460 analyzer (Micromeritics, GA, USA) with ultra-high purity N_2_ gas. The amounts of ReCC loaded onto the samples were detected by Agilent ICP-OES730 (agilent, Palo Alto, CA, USA). Fourier Transform Infrared Spectrometer (FT-IR) spectra were monitored by the iS10 FT-IR spectrometer (Thermo Fisher Scientific, Waltham, MA, USA). X-ray photoelectron spectroscopy (XPS) measurements were carried out using a Thermo Scientific Esca Lab 250Xi (Waltham, MA, USA) with a 900 mm spot size in an ultra-high vacuum chamber, The pass energy was 20 eV. The thermal analysis (TG) instrument model utilized was the Netzsch STA 449 F3/F5 (Netzsch, Selb, Germany). The gas chromatography detection was performed by a Fuli GC 9790plus instrument, where the gas phase separation column used was TDX-01 (Thermo Fisher Scientific, Waltham, MA, USA). An electrochemical test was performed on the Netherlands Ivium-n-Stat electrochemical analyzer. The electrochemical impedance spectroscopy (EIS) measurements, Mott–Schottky curves, and the photocurrent response were performed on the electrochemical analyzer with a three-electrode cell. The electrolyte was 0.25 M Na_2_SO_4_ solution. To prepare the working electrolyte, catalysts were added to the solution of Nafion in ethanol and then sonicated and dropped onto the FTO conductive glass, where the working effective area was 0.25 cm^2^. An Ag/AgCl electron was used as a reference electrode, and the platinum plate worked as a counter electrode. Similarly, cyclic voltammograms (CVs) were recorded on the Netherlands Ivium-n-Stat electrochemical analyzer with a platinum plate as the working electrode, Ag/AgCl as the reference electrode, and Pt wire as the counter electrode using 0.1 M tetrabutylammonium tetrafluoroborate (TBAPF_6_) as a supporting electrolyte in DMF. The Mott–Schottky curves were tested for two different frequencies (500 and 1000 HZ).

### 3.4. Photocatalytic CO_2_ Reduction Test

A 10 mg photocatalyst sample and 134 mg BIH as sacrificial agent were added into 5 mL DMF in a quartz cell (30 mL total volume) sealed with a septum, vacuumed, and bubbled with CO_2_ 3 times (0.5 h of CO_2_ bubble time for every treatment process) and then illuminated with a xenon lamp (300 W) equipped with a cutoff filter for visible light (λ > 420 nm). The quantity of CO production and other substances in the reaction cell above were detected by gas chromatography (GC) using a TCD and FID detectors (Fuli Model GC9790 II) (Fuli, Zhejiang China). To evaluate the photocatalytic stability performance of the hybrid material, the photocatalytic CO_2_ reduction long-durability test for ReCC-TiO_2_-5 wt% and ReCC@TiO_2_-5 wt% samples was performed for 15 h to confirm the stability performance of the hybrid in this work. A transient photocurrents test was carried out per 40 s as one cycle and 20 s as one node, along with turning on and off the light to test its photoelectric response ability for a total duration of 400 s.

## 4. Conclusions

We designed and prepared a robust porous composite material ReCC-TiO_2_ based on the ReCC complex and anatase TiO_2_ by a facile sol–gel method. Compared with the sensitization system ReCC@TiO_2_, the robust coordination linkage between ReCC and TiO_2_ in the hybrid provided much higher CO_2_ photoreduction activity and more outstanding stability under similar conditions. Among them, the ReCC-TiO_2_ hybrid materials doped with 5.0 wt% ReCC complex exhibited a great TON_CO_ value of 721 (based on the mass of ReCC) in the DMF system with BIH as a sacrificial agent. The excellent performance of the ReCC-TiO_2_ composites can be attributed to the synergistic effect and the more efficient charge separation and electron transfer between ReCC and TiO_2_. In particular, the effective combination of ReCC with TiO_2_ could promote the capability of recycled utilization for ReCC. It further revealed that the TiO_2_ inorganic substrate possesses the functions of supporting, dispersing, and protecting other components in the hybrid. The mutual achievement of ReCC and TiO_2_ provides a potential approach to build robust and active organic–inorganic functional composites for an efficient photocatalysis process.

## Data Availability

The data presented in this article are available on request from the corresponding author.

## Supplementary Materials

The following supporting information can be downloaded at: https://www.mdpi.com/article/10.3390/ijms241311106/s1.
