# Supplementary material for: The Facile Synthesis of a Re-Complex Heterogeneous Catalysis System for Enhancing CO2 Photoreduction Activity
Source: Int J Mol Sci. 2023 Jul 5;24(13):11106. doi: 10.3390/ijms241311106 (PMC10342104; doi:10.3390/ijms241311106)
Supplement: Supplementary file 1 [file ijms-24-11106-s001.zip › ijms-2458825-supplementary.pdf]

Supplementary Information

The facile synthesis of Re-complex heterogeneous catalysis system for enhancing photoreduction CO_2 activity

Bo Li, Hang Li, Shiyan Liang, Xin Zhong, Jiaao Cheng, Yifan Chen* and Yujie Song*

[1]Hainan Provincial Key Laboratory of Fine Chemicals, College of Chemical Engineering and Technology, Hainan University, Haikou, China

*** Correspondence:**
Yi-Fan Chen
chenyifan@hainanu.edu.cn

Yujie Song
songyuejie@hainanu.edu.cn

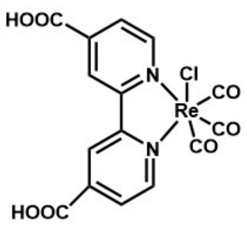

ReCC

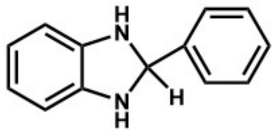

BIH

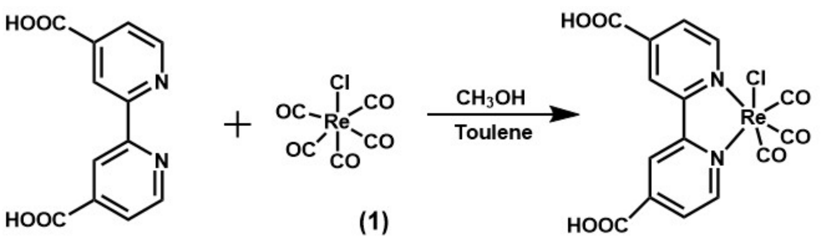

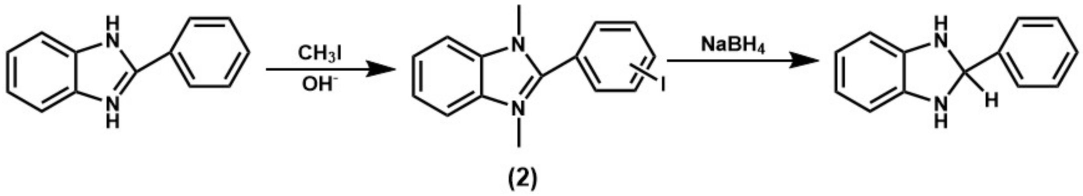

Scheme S1 the structure and synthesis route of ReCC and BIH.

Table S1 Specific surface area, pore size and pore volume of different material s

Sample	Surace area (m^2/g)	Pore size（nm）	Pore volume (nm)
Gel-TiO_2	231.3	4.54	0.262
ReCC-TiO_2-5wt%	258.1	3.96	0.255

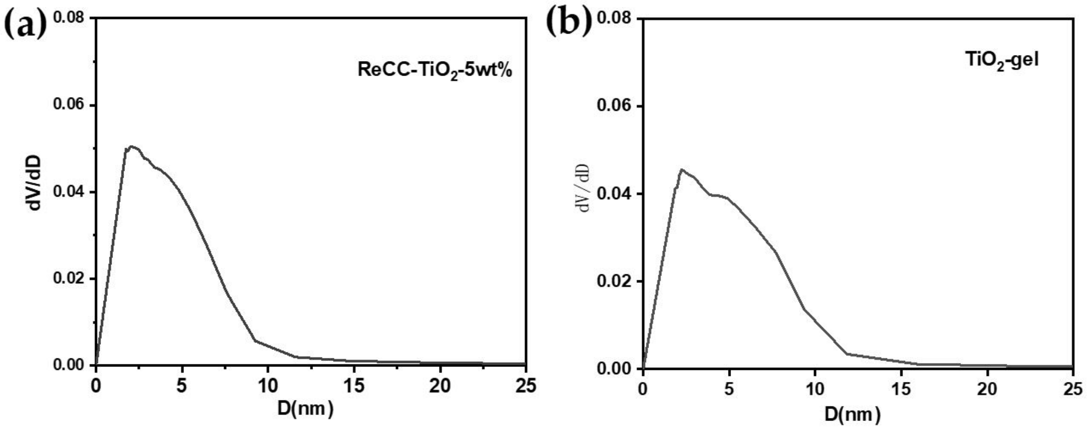

Figure S1. ReCC-TiO_2-5wt% (a) and TiO_2 (b) the quantity distribution of pore size of.

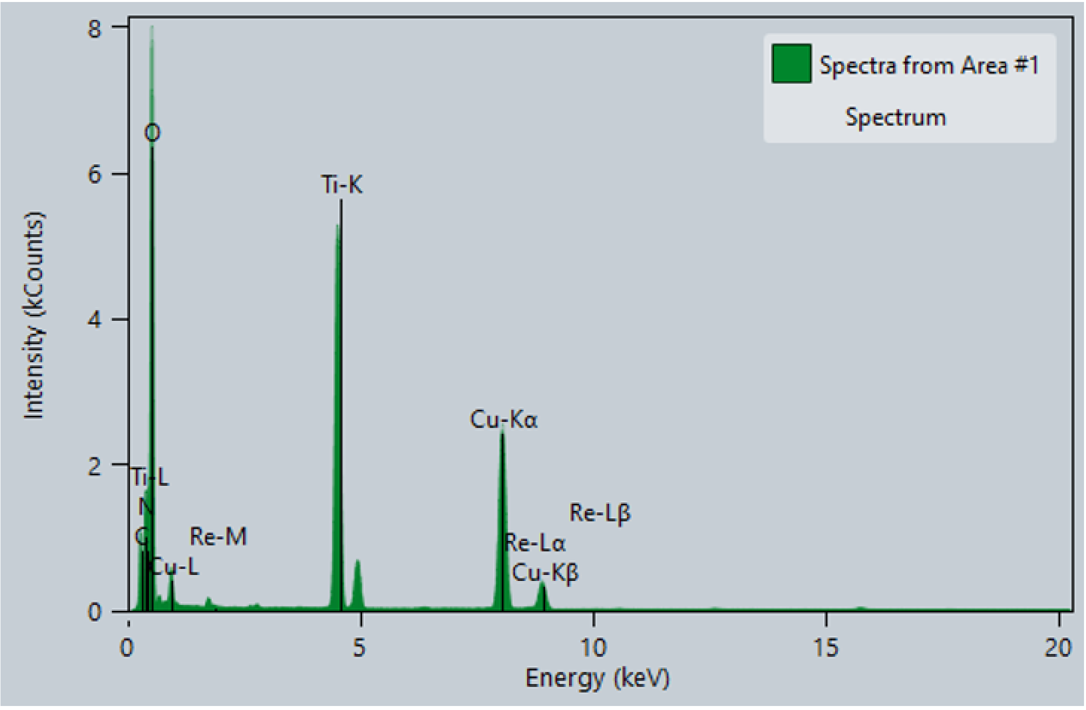

Figure S2. Energy diagram and the atomic fraction of ReCC-TiO_2-5wt%.

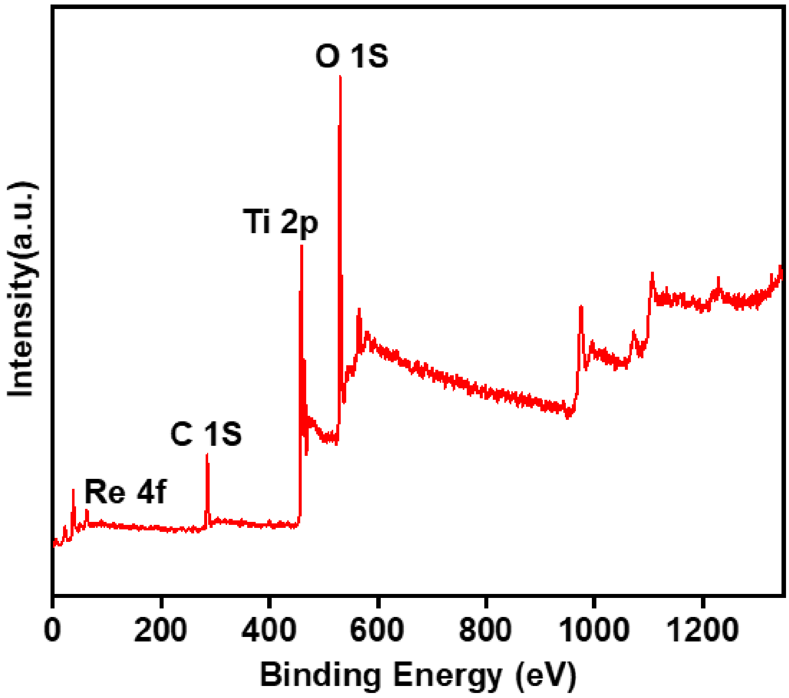

Figure S3. XPS survey spectrum of ReCC-TiO_2-5wt%.

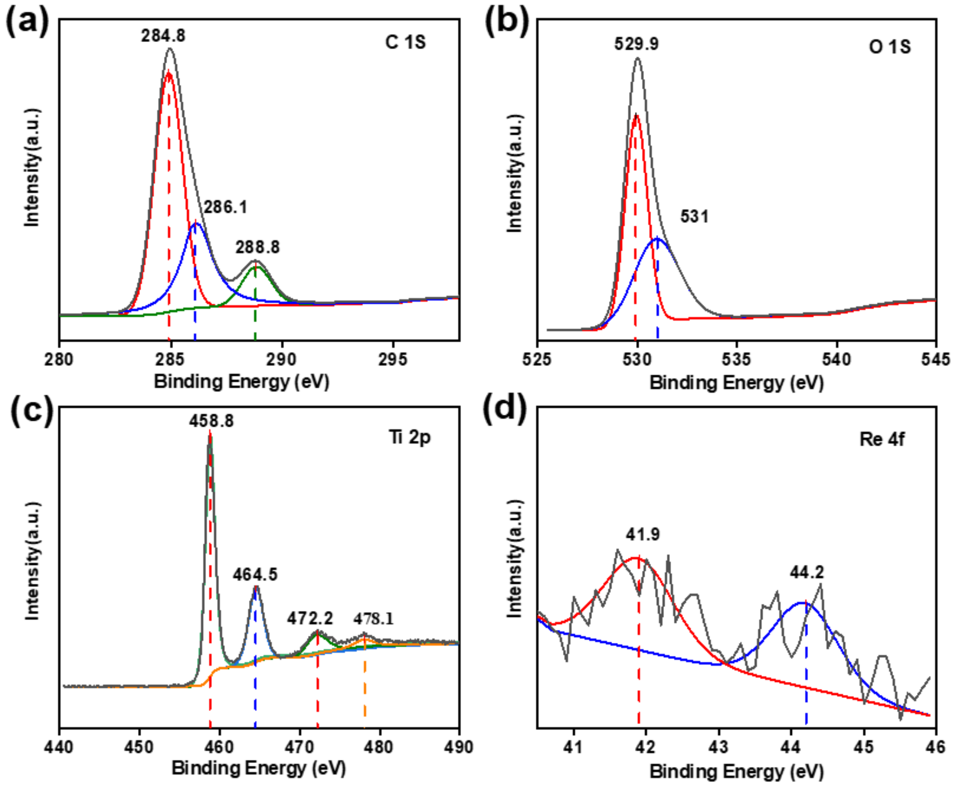

Figure S4. (a, b, c and d) high-resolution XPS spectra of C 1s, O 1s, Ti 2p and Re 4f for ReCC-TiO_2-5wt%

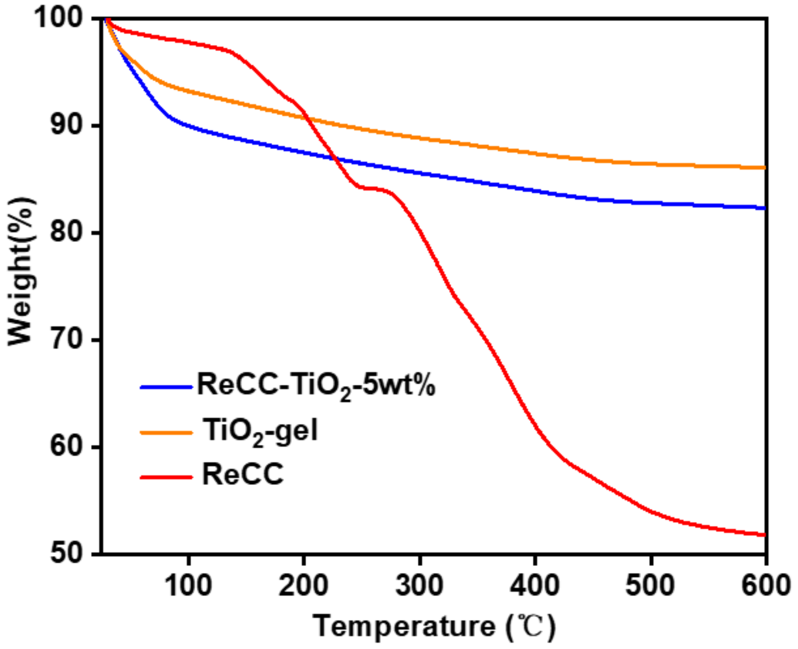

Figure S5. Thermogravimetric analysis of ReCC,TiO_2 and ReCC-TiO_2-5wt%.

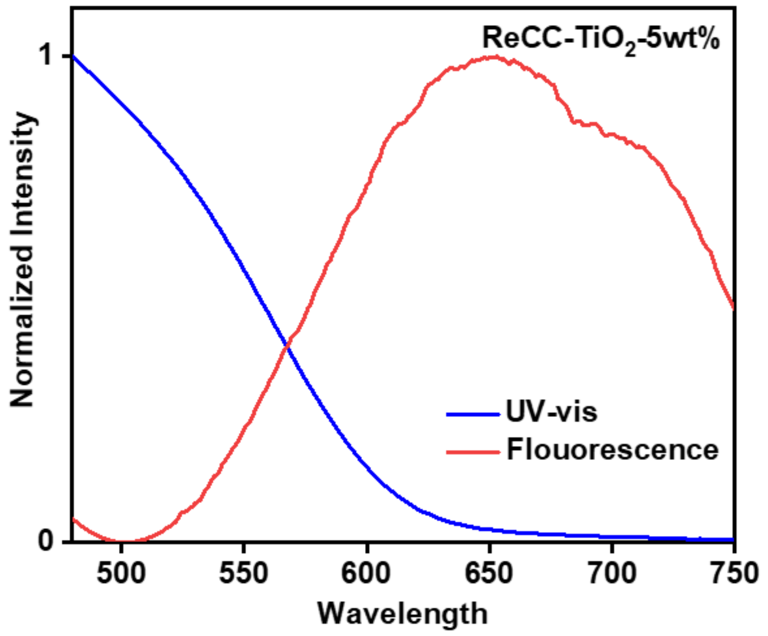

Figure S6. Uv-vis spectrum and fluorescence emission spectrum of ReCC.

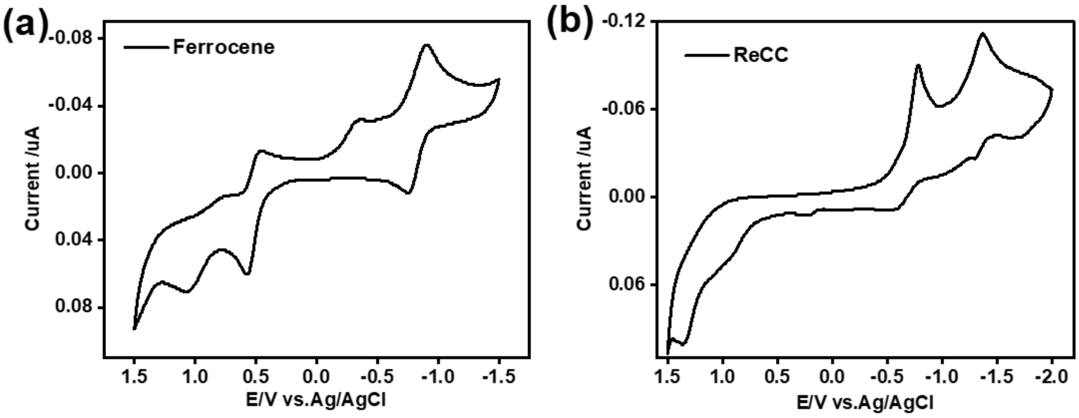

Figure S7. Electrochemical cyclic voltammetry test diagram of ferrocene (a) and ReCC (b)

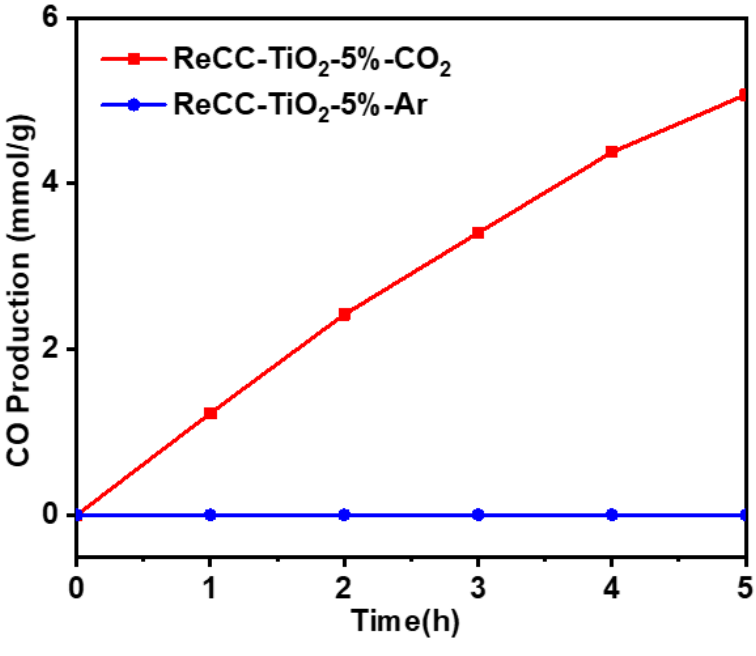

Figure S8. The samples on the photoreduction activity for CO production in 10 mL DMF with 134 mg BIH as the electron donor under visible light irradiation ($\lambda > 420$ nm) of ReCC-TiO_2-5wt%-CO_2 and ReCC-TiO_2-5wt%-Ar.

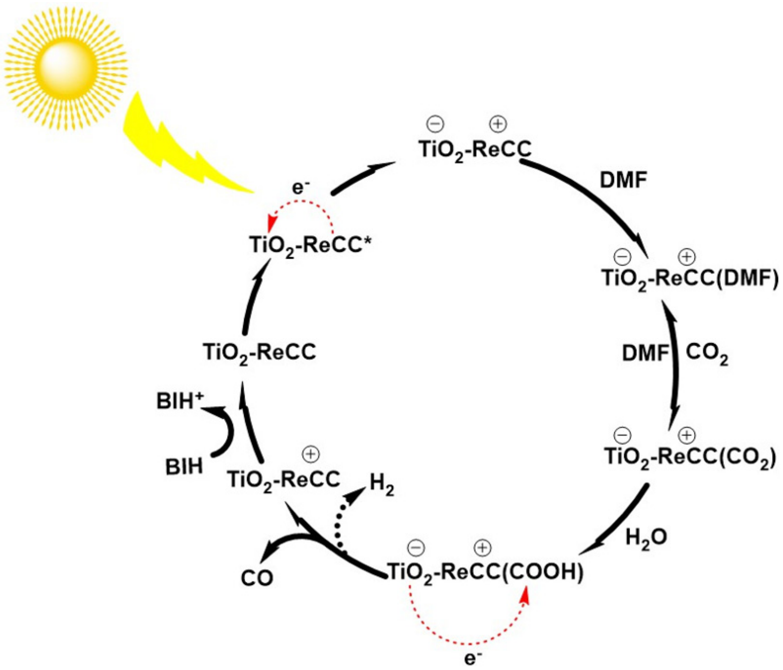

Figure S9. Possible mechanism of photoreduction CO_2 for CO production over ReCC-TiO_2.

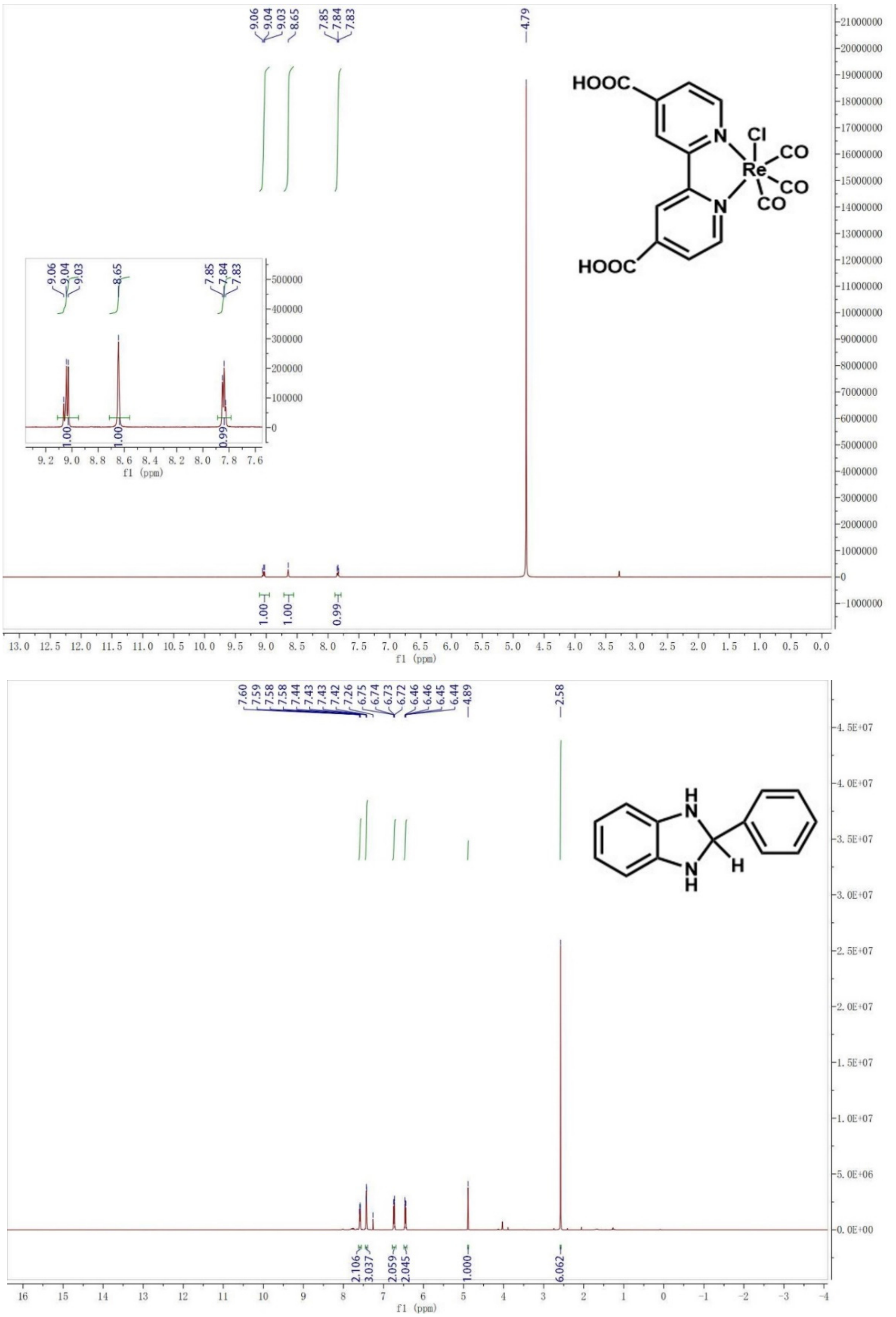

Figure S10. NMR spectra of ReCC and BIH.
